# Vegetation affinity of species *Typha shuttleworthii* in the western part of the Carpathians, with *Typhetum shuttleworthii* as a new association to Slovakia

**DOI:** 10.3897/BDJ.8.e52151

**Published:** 2020-05-04

**Authors:** Richard Hrivnák, Michal Slezák, Drahoš Blanár, Pavel Širka, Kateřina Šumberová

**Affiliations:** 1 Institute of Botany, Plant Science and Biodiversity Center, Slovak Academy of Sciences, Bratislava, Slovakia Institute of Botany, Plant Science and Biodiversity Center, Slovak Academy of Sciences Bratislava Slovakia; 2 Slovak Academy of Sciences, Zvolen, Slovakia Slovak Academy of Sciences Zvolen Slovakia; 3 Podtatranské Museum in Poprad, Poprad, Slovakia Podtatranské Museum in Poprad Poprad Slovakia; 4 Administration of the Muránska planina National Park, Revúca, Slovakia Administration of the Muránska planina National Park Revúca Slovakia; 5 Department of Phytology, Faculty of Forestry, Technical University in Zvolen, Zvolen, Slovakia Department of Phytology, Faculty of Forestry, Technical University in Zvolen Zvolen Slovakia; 6 Department of Vegetation Ecology, Institute of Botany of the Czech Academy of Sciences, Brno, Czech Republic Department of Vegetation Ecology, Institute of Botany of the Czech Academy of Sciences Brno Czech Republic

**Keywords:** Europe, threatened species, wetland vegetation

## Abstract

*Typha shuttleworthii* (Shuttleworth’s bulrush) is a rare species throughout its distribution range including Carpathians. However, a substantial increase in its finds has been noticed in the last twenty years. This study summarises the present knowledge and brings new data on vegetation with *T. shuttleworthii* occurrence from the western part of the Carpathians (Czech Republic, Slovakia, Poland and Ukraine) with the aims of evaluating the phytosociological affinity of this species and providing new information about the ecology of the relevant plant communities. We found that *T. shuttleworthii* mainly occurred in marsh vegetation (the *Phragmito-Magnocaricetea* class) including the *Typhetum shuttleworthii* association. Some plots also corresponded to transitional stands between marshes and wet meadows of the *Molinio-Arrhenatheretea* class (*Molinietalia caeruleae* order). Moisture and soil reaction were identified as principal factors responsible for variation in species composition of the vegetation. *Typhetum shuttleworthii* was recognised as new for the territory of Slovakia and confirmed in all other countries, Czech Republic, Poland and Ukraine. Our results could contribute to better preservation of the species and its habitats and thus be very important for practical nature conservation.

## Introduction

Freshwater habitats, such as shallow field and meadow depressions, ponds or river arms, are very dynamic ecosystems and cover an important part of plant diversity, including endangered and rare plant species (Lukács et al. 2013, Biggs et al. 2017, Bubíková and Hrivnák 2018). Vegetation ecology research of these habitats in the Carpathian region of Central and Eastern Europe permanently brings new and interesting findings. *Typha shuttleworthii* and *Typhetum shuttleworthii*, as a community in which it dominates, are typical examples. *Typha shuttleworthii* (Shuttleworth's bulrush) is a robust perennial plant recorded in Central and Southern Europe, from eastern France to Ukraine, Romania and Bulgaria, as well as in Turkey and Iran in south-western Asia (cf. Kozłowska et al. 2011, Nobis et al. 2015). Shuttleworth's bulrush has a scattered occurrence in the whole Carpathians and it was recently recorded in the western part of the mountains as new to Poland (Kozłowska et al. 2011), while, in the last two decades in several other countries, such as the Czech Republic, Slovakia, Ukraine and Romania, the number of records about its occurrence also increased (e.g. Mânzu and Chifu 2003, Uhrin and Bača 2005, Bartošová et al. 2008, Felbaba-Klushina 2009, Felbaba-Klushina 2009, Borsukevych 2011). Recent knowledge about phytosociology of the species in the Carpathians showed its presence in wetland communities arranged into *Phragmito-Magnocaricetea*, *Scheuchzerio palustris-Caricetea fuscae* and *Molinio-Arrhenatheretea* (e.g. Felbaba-Klushina 2009, Lukács et al. 2013, Kozłowska et al. 2011). *Typhetum shuttleworthii*, as a marsh plant community with strong dominance of Shuttleworth's bulrush, is documented only by a few relevés from the Czech Republic, Slovenia, Romania and Ukraine (Nedelcu et al. 1979, Vreš et al. 2001, Borsukevych 2011, Šumberová et al. 2011, Landucci et al. 2020, Dubyna et al. 2020) and only a part of them was recorded in the Carpathians (e.g. Mânzu and Chifu 2003, Šumberová et al. 2011). However, the number of localities of the species is much higher than occurrences of the association. Similarly in Slovakia, *Typha shuttleworthii* was found in a few localities (Dostál and Červenka 1992, Ondrášek 2002, Dubyna et al. 2020, Uhrin and Bača 2005), while vegetation with its occurrence was documented only by one relevé (Uhrin and Bača 2005) without a clear syntaxonomical classification, only as meadows with extensive management. Several new localities have recently been found in the country (Veverka 2018, Hrivnák et al. 2019) and relevant vegetation was recorded.

Therefore, we would like (1) to evaluate the vegetation affinity of the species *T. shuttleworthii* and (2) to provide new information about the ecology of relevant vegetation types within the western part of Carpathians.

## Material and methods

**Figure 1.**
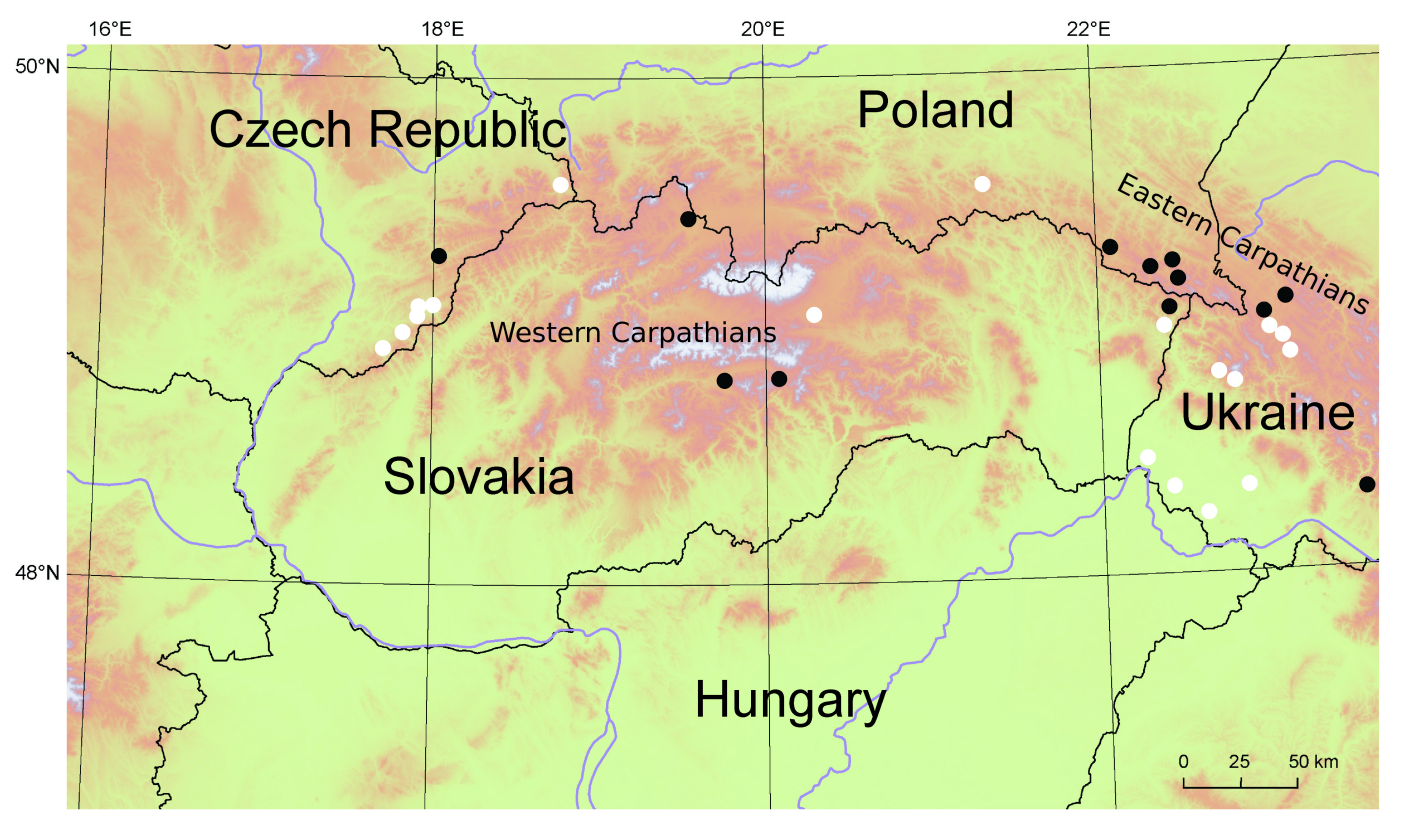
Position of phytosociological relevés and floristic data of *Typha shuttleworthii* used in our study in the western part of the Carpathians (black circles – phytosociological relevés and white circles – floristic data).

Vegetation, with the presence of *T. shuttleworthii*, was studied using both the traditional Zürrich-Montpellier´s approach (Westhoff and van der Maarel 1973) and the Braun-Blanquet scales (old and new with transforming into the old scale in phytosociological tables; Suppl. material 1) in the western part of the Carpathian Mts. characterised by a typical (sub)montane climate and a heterogeneous geological bedrock, consisting of both calcareous and non-calcareous substrates. Phytosociological relevés were obtained from the western part of Carpathians (Czech Republic, Poland, Slovakia and Ukraine). In addition to the already published phytosociological material (Uhrin and Bača 2005, Bartošová et al. 2008, Borsukevych 2011, Kozłowska et al. 2011), five unpublished relevés from three localities in Slovakia were recorded in 2016 and 2018 (Fig. 1). Altogether, 22 relevés were stored in a TURBOVEG database (Hennekens and Schaminée 2001), exported and processed in the Juice programme (Tichý 2002). Species nomenclature and taxonomy were in some cases unified, using broadly defined plant taxa: *Agrostis stolonifera* agg. (incl. *A. gigantea* and *A. stolonifera*), *Dactylorhiza majalis* s. lat. (*D. majalis* and *D. maculata*), *Eleocharis palustris* agg. (*E. palustris* and *E. uniglumis*) and *Myosotis palustris* agg. (*M. laxiflora* and *M. palustris*). Then, the dataset was classified in the PC-ORD programme (McCune and Mefford 1999), using the relative Sorensen distance as a dissimilarity measure and the Beta flexible linkage method (*ß* = –0.25) with the logarithmic data transformation. This classification was done with the aim of showing the main vegetation types with the occurrence of *T. shuttleworthii*. Diagnostic species of each cluster were identified, using both frequency and fidelity thresholds (Φ – phi coefficient; Chytrý et al. 2002) and fidelity calculation followed presence/absence data with standardisation of relevé groups to an equal size. The Fisher’s exact test (*p* < 0.05) was used to eliminate species with non-significant occurrence in a particular cluster (Tichý and Chytrý 2006). Subsequently, diagnostic species were defined as species showing simultaneously frequency ≥ 20%, phi coefficient ≥ 0.50 and difference in frequencies amongst clusters ≥ 20%. If a particular species were constant (frequency ≥ 50%) in two or more clusters, it was not accepted as diagnostic. In addition, the classification approach for the identification of European marsh vegetation of the *Phragmito-Magnocaricetea* class and the formal definition of *Typhetum shuttleworthii* was used only for the identification of the *Typhetum shuttleworthii* association (Landucci et al. 2020).

Vegetation-environmental relationships were analysed using Detrended Correspondence Analysis (DCA) in the Canoco for Windows package (ver. 5.0; ter Braak and Šmilauer 2012). DCA ordination was performed with detrending by segments and logarithmic transformation of species cover values. Ecological gradients affecting variation in species composition were interpreted by unweighted means of Ellenberg indicator values for vascular plants (EIVs for continentality, light, moisture, nutrients, soil reaction and temperature; Ellenberg et al. 1992), which were inserted into an ordination diagram as supplementary variables. Correlations between the positions of relevés along the first two axes and the given EIV per relevé were calculated as weighted (weight = case total) Pearson correlation (further *r*).

Soil samples were taken from the uppermost mineral horizon (0–10 cm depth, litter removed) only in Slovak localities. They were dried at laboratory temperature, crushed and passed through a 2 mm sieve. Soil pH and conductivity were measured in a distilled water solution (soil/water ratio of 1/5) using Eutech Instruments PC 650. In addition, altitude, mean annual temperature and total annual precipitation were detected in Slovak localities and they are presented in Suppl. material 1. Altitude was measured with a GPSmap 60 CSx device in the field. Climatic characteristics, derived from GIS layers, were provided by GeoModel Solar Company (GeoModelSolar 2014).

Nomenclature of vascular plant species and communities followed Marhold and Hindák 1998, Mucina et al. 2016 and Jarolímek and Šibík 2008 with the exception of *Typhetum shuttleworthii* Nedelcu et al. ex. Šumberová in Chytrý 2011. Nomenclature of bryophytes is according to Mišíková et al. 2020.

## Results

Numerical classification of phytosociological relevés with *Typha shuttleworthii* distinguished three clusters (Table 1, Suppl. material 1). The first cluster represents species-poor (~ 5) and very heterogeneous vegetation, poorly differentiated by diagnostic species from the other two clusters. All relevés were assigned to the *Phragmito-Magnocaricetea* class. The second cluster is characterised by a relatively homogenous species composition with an average species richness of ~ 14 and is well differentiated from the other two clusters by a relatively high number of diagnostic species of marshes (*Phragmito-Magnocaricetea* class), as well as of fens (*Scheuchzerio palustris-Caricetea fuscae* class). However, both relevés of this cluster were recorded at the same locality and assigned to the *Phragmito-Magnocaricetea* class. The third cluster contains the highest number of relevés, vegetation is relatively species rich (~ 19) and the diagnostic species group consisted mainly of species related to marshes (*Phragmito-Magnocaricetea*) and wet meadows (*Molinietalia caeruleae* order of the *Molinio*-*Arrhenatheretea* class). These relevés were assigned to the *Phragmito-Magnocaricetea* class, but several of them showed a transitional position between both mentioned vegetation units and in one case, *Salix caprea* was the dominant species in the shrub layer.

**Figure 2.**
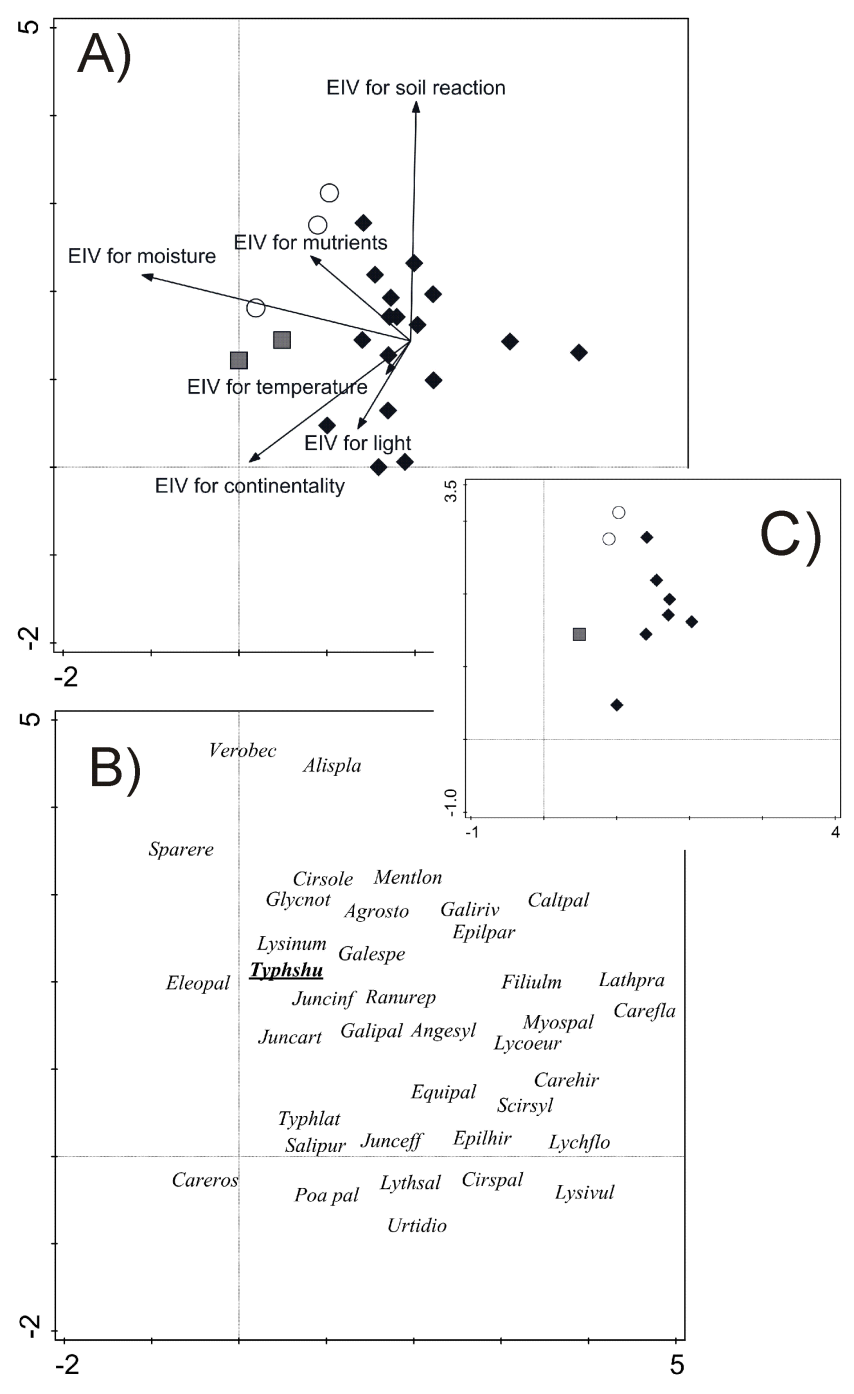
Detrended Correspondence Analysis of relevés with *Typha shuttleworthii* occurrence; A) relevés (empty circles = cluster 1, shaded squares = cluster 2 and black diamonds = cluster 3) and environmental variables represented by species’ Ellenberg indicator values; B) species with presence in at least 4 relevés (≥ 18% of all relevés) and C) relevés of *Typhetum shuttleworthii*. Abbreviations of species presented in the ordination diagram: Agrosto – *Agrostis stolonifera*, Alispla – *Alisma plantago-aquatica*, Angesyl – *Angelica sylvestris*, Caltpal – *Caltha palustris*, Carefla – *Carex flava* agg., Careros – *Carex rostrata*, Cirsole – *Cirsium oleraceum*, Cirspal – *Cirsium palustre*, Eleopal – *Eleocharis palustris* agg., Epilhir – *Epilobium hirsutum*, Epilpar – *Epilobium parviflorum*, Filiulm – *Filipendula ulmaria*, Galespe – *Galeopsis speciosa*, Galipal – *Galium palustre*, Galiriv – *Galium rivale*, Glycnot – *Glyceria notata*, Junceff – *Juncus effusus*, Juncinf – *Juncus inflexus*, Lathpra – *Lathyrus pratensis*, Lychflo – *Lychnis flos-cuculi*, Lycoeur – *Lycopus europaeus*, Lysinum – *Lysimachia nummularia*, Lysivul – *Lysimachia vulgaris*, Lythsal – *Lythrum salicaria*, Mentlon – *Mentha longifolia*, Myospal – *Myosotis palustris* agg., Poa pal – *Poa palustris*, Ranurep – *Ranunculus repens*, Salipur – *Salix purpurea*, Scirsyl – *Scirpus sylvaticus*, Sparere – *Sparganium erectum*, Typhlat – *Typha latifolia*, Typhshu – *Typha shuttleworthii*, Urtidio – *Urtica dioica*, Verobec – *Veronica beccabunga*.

**Figure 3.**
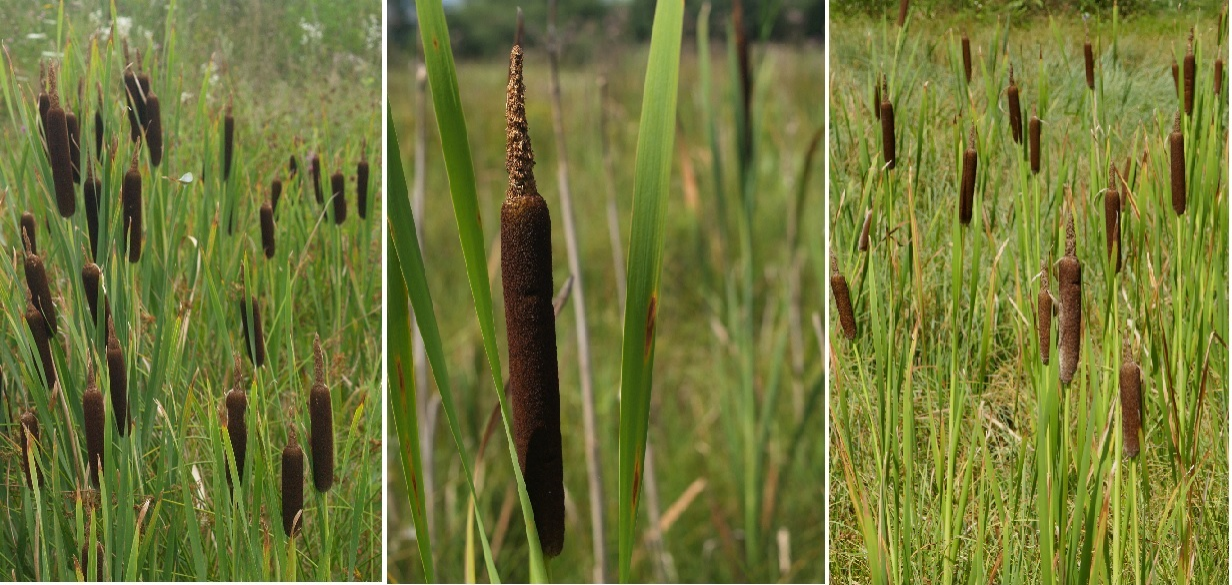
Stands of the *Typhetum shuttleworthii* association in Slovakia (Rohozná: author R. Hrivnák, 24. 7. 2018; Bobrov, R. Hrivnák, 2. 8. 2016; Červená Skala, D. Blanár, 24. 8. 2018; from left to right).

The cover of *Typha shuttleworthii* in vegetation patches is very variable (Suppl. material 1). All clusters are relatively-well distinguished in ordination space (Fig. 2A) with distinct species composition (Fig. 2B). *Typhetum shuttleworthii*, as a marsh community, was identified in all clusters Suppl. material 1; Fig. 2C and Fig. 3).

Altogether, the first two DCA axes explained 18.5% and 25.8% variability in species data and species-environment relationship, respectively. EIV for moisture was identified as the environmental variable strongly correlated with the first DCA axis (*r* = 0.72), while EIV for soil reaction was the most correlated variable with the second DCA axis (*r* = 0.61). Clusters are arranged along the first DCA axis in the following order: 3→1→2 and show preferences for increased EIV for moisture, continentality and nutrients. Species are grouped from moisture- and nutrient-demanding marsh taxa such as *Carex rostrata*, *Eleocharis palustris* agg., *Sparganium erectum* or *Veronica beccabunga* to taxa of wet meadows, such as *Carex flava* agg., *Lathyrus pratensis* or *Myosotis palustris* agg. (Fig. 2B). On the contrary, there is an obvious sequencing only in the case of the first and second cluster (2→1) along the second DCA axis represented by EIV for soil reaction, while the third cluster is very heterogeneous along this gradient (Fig. 2A). The position in the ordination space suggests that *Typha shuttleworthii* prefers marshy and nutrient rich habitats with moderate values of soil reaction (Fig. 2B). Directly measured characteristics, both soil reaction and conductivity, showed similar habitat preferences of the species, from slightly acidic to neutral soil reaction and moderate values of conductivity (Table 2).

## Discussion

The results of our study confirmed that vegetation with the presence of *Typha shuttleworthii* is relatively heterogeneous in the western part of the Carpathian Mts. *Typha shuttleworthii* occurs in marshes (*Phragmito-Magnocaricetea* class) and, in several cases, stands had a transitional position between marshes and wet meadows (*Molinio-Arrhenatheretea* class, *Molinietalia caeruleae* order) with the mosaic of marshy, wet meadows and fen species. Similar evaluation of *T. shuttleworthii* coenology is known from some other European countries. For example, in the Ukrainian Eastern Carpathians and the adjacent regions, *T. shuttleworthii* grew in marshes and fens belonging to the alliances *Phragmition communis* (*Phragmito-Magnocaricetea*) and *Caricion atrofusco-saxatilis*, *Caricion davallianae* (*Scheuchzerio palustris-Caricetea fuscae*; Felbaba-Klushina 2009, Felbaba-Klushina 2009). Shuttleworth's bulrush grew in the marshy littoral zone of small forest ponds (*Phragmito-Magnocaricetea*) and wet meadows with the dominance of *Scirpus sylvaticus* (*Calthion palustris* alliance, *Molinio-Arrhenatheretea*) in the central part of the Czech Republic (Hlaváček 2009, Šumberová et al. 2011). In addition to natural habitats, stands with *T. shuttleworthii* were also found in typical anthropogenic habitats such as roadside ditches, rills or waterlogged pits in quarries (Kozłowska et al. 2011, Nobis et al. 2015).

Our results showed preferences of *T. shuttleworthii* to marshy and nutrient-rich habitats. Results are in accordance with known data; *T. shuttleworthii* was recorded in mesotrophic to eutrophic habitats, periodically or permanently waterlogged by shallow water (Nedelcu et al. 1979, Vreš et al. 2001, Šumberová et al. 2011). Soil reaction from Slovak localities was slightly acidic to neutral, similarly to the values recognised in Romania and Slovenia with pH 6.2–7.1 and 5.9–7.8 (rarely 4.0), respectively (Nedelcu et al. 1979, Vreš et al. 2001).

Only stands with a high cover of *Typha shuttleworthii* and a significant portion of marshy species at the expense of meadow herbs were assigned to *Typhetum shuttleworthii* association (*Phragmition communis* alliance) in our study. While this association has been reported from several adjacent countries, namely the Czech Republic, Romania and Ukraine (Borsukevych 2011, Šumberová et al. 2011, Zamfirescu and Zamfirescu 2006, Dubyna et al. 2020), it has not yet been presented from the territory of Slovakia. In addition, despite a few published relevés about the presence of *T. shuttleworthii* in Poland (Kozlowska 2011), *Typhetum shuttleworthii* is, for the first time, detected for this country in our study. *Typhetum shuttleworthii* is a relatively rare and poorly-documented plant community in the whole of Europe (Landucci et al. 2020). This fact is, in our opinion, related to (1) a scarce and scattered occurrence of dominant species *Typha shuttleworthii* in the European territory (https://www.gbif.org/species/5289496), (2) forming, in most cases, only small patches or the situation when individual plants/clones are dispersed in other vegetation types and (3) relatively-poor competitive ability of the target species compared to other species of the *Typha* genus, for example, *T. latifolia* (Kapitonova et al. 2015). *T. shuttleworthii* probably differs, at least partly, from the other *Typha* species occurring in central Europe by its habitat preferences. Based on the studied vegetation types with *T. shuttleworthii* occurrence, it can be concluded that this species prefers habitats rich in mineral nutrients, which are, however, not affected by strong eutrophication. The substantially narrower ecological range differentiates *T. shuttleworthii* from the common *T. latifolia*, the species very common in hypertrophic habitats, but also able to grow in wetlands with rather limited amounts of nutrients (Šumberová et al. 2011). While *T. shuttleworthii* often grows in disturbed habitats without a developed humus horizon, *T. latifolia* frequently occurs in water bodies with a thick layer of sapropelic mud on the bottom. Similar affinity to disturbed habitats as *T. shuttleworthii* shows *T. laxmannii*, the bulrush species considered as non-native in central Europe and invasive in some of its parts. *T. laxmannii*, however, exhibits a high ability to spread quickly and it often colonises large areas mainly in artificial habitats, such as sand pits, canals or water reservoirs within a short time (Nobis et al. 2006, Oťaheľová et al. 2001). It also well tolerates sub-saline conditions and thus often occurs in artificial habitats under the influence of winter salting, such as roadside ditches, village ponds and waste places in the settlements (Kaplan et al. 2019). There are no comparative ecological studies of European bulrush species, based on the analysis of a large amount of data, including the results of soil physical and chemical properties. Therefore, it is impossible to develop our ecological evaluation of *T. shuttleworthii* in more detail.

Most of the available observations of *Typha shuttleworthii* occurrence in Central Europe originate mainly from the last two decades (e.g. Hlaváček 2009, Grulich 2009, Borsukevych 2011, Kozłowska et al. 2011, Veverka 2018). Obviously, this easily-recognisable species did not occur in these localities because old and mostly complex floristic papers published from particular areas in the past did not record it (cf. Hlaváček 2009). The same is also true at least for one of the Slovak localities near Červená Skala in the Muránska planina Mts. (Hrivnák et al. 2019). Intensive floristic research, conducted there in the past (Hendrych 1969, Kochjarová et al. 2004), did not detect the occurrence of *T. shuttleworthii*. Stands recorded from a small carst pool in 2001 were assigned to fen vegetation of *Caricetum goodenowii* (*Caricion fuscae* alliance; see Suppl. material 2 and Hrivnák et al. 2008) without the presence of *T. shuttleworthii*, but with *T. latifolia* (cover less than 1%). The recently recorded vegetation (Suppl. material 1, rels 4–5) is, however, very different.

*Typha shuttleworthii* is a rare species in several Carpathian countries. The species is critically endangered in Slovakia and the Czech Republic (Grulich 2012, Eliáš et al. 2015), endangered in the Ukrainian Carpathians (Kricsfalusy and Budinkov 2007) and vulnerable in Poland (Kozłowska et al. 2011). Similarly, it was evaluated as a vulnerable species for the whole Carpathian Mts. (Turis et al. 2014). Preservation of the species and its habitats is thus very important from the nature conservation point of view. Protection measures should include, amongst others, legislative protection of populations and stands "in situ", but at the same time, preservation of the water regime of *T. shuttleworthii* localities, as well as regulation of human activities in their surroundings, particularly those leading to eutrophication. However, specific ecological conditions of the species, its frequent occurrence in pioneer, ecologically very sensitive vegetation and/or artificial habitats with strong natural successional and/or human impacts (e.g. direct destruction or draining), as well as on small patches, are unfavourable for practical conservation. Therefore, the conservation of the species and its community should not be generalised and it must be specifically tailored with regard to the specific conditions of individual sites.

## Supplementary Material

EBD5CDB9-2E97-558E-84B3-73D81705AD21 10.3897/BDJ.8.e52151.suppl1 Supplementary material 1 Shortened full table of phytosociological relevés Data type phytosociological relevés File: oo_402707.doc https://binary.pensoft.net/file/402707 Richard Hrivnák, Michal Slezák, Drahoš Blanár, Pavel Širka, Kateřina Šumberová

672251D5-231A-5223-AEA3-CC54D2E85951 10.3897/BDJ.8.e52151.suppl2 Supplementary material 2 Unpublished phytosociological relevé Data type phytosociological relevé File: oo_402706.docx https://binary.pensoft.net/file/402706 Drahoš Blanár

## Figures and Tables

**Table 1.** Shortened synoptic table of phytosociological relevés with the occurrence of *Typha shuttleworthii* in the Western Carpathians and the adjacent part of the Eastern Carpathians. Only species with occurrence in at least 3 relevés are presented. Unpublished Slovak relevés with all plant taxa are stored in national databases GIVD ID: EU-SK-001 (http://ibot.sav.sk/cdf/). *MA – *Molinio-Arrhenatheretea*, PM – *Phragmito-Magnocaricetea*, SC – *Scheuchzerio-Caricetea fuscae* species. Shortened full table and localities of relevés are presented in Suppl. material 1.

*	**Number of all relevés in cluster**	**3**	**2**	**17**
	**Share of relevés from Poland in the cluster (%)**	**33**	.	**47**
	**Share of relevés from Slovakia in the cluster (%)**	.	**100**	**24**
	**Share of relevés from Ukraine in the cluster (%)**	**67**	.	**29**
	*Typha shuttleworthii*	100	100	100
	**Diagnostic species of first cluster**			
	*Equisetum arvense*	33	.	.
	**Diagnostic species of second cluster**			
SC	*Calliergonella cuspidata*	.	100	.
	*Drepanocladus aduncus*	.	100	.
SC	*Calliergon giganteum*	.	100	.
	*Cardamine hirsuta*	.	100	.
PM	*Sparganium erectum*	33	100	6
	*Juncus articulatus*	.	100	18
PM	*Carex rostrata*	.	100	18
	**Diagnostic taxa of third cluster**			
PM	*Juncus effusus*	.	.	71
PM	*Lycopus europaeus*	.	.	65
PM, MA	*Lythrum salicaria*	.	.	53
MA	*Agrostis stolonifera* agg.	.	.	47
PM	*Epilobium hirsutum*	.	.	47
	*** Phragmito-Magnocariceta ***			0
	*Galium palustre*	.	100	41
	*Typha latifolia*	33	50	35
	*Glyceria notata*	33	.	29
	*Eleocharis palustris* agg.	.	100	24
	*Alisma plantago-aquatica*	33	.	12
	***Molinio-Arrhenatheretea***			
	*Mentha longifolia*	100	.	71
	*Myosotis palustris* agg.	.	100	53
	*Scirpus sylvaticus*	.	.	59
	*Filipendula ulmaria*	.	.	41
	*Galium rivale*	.	.	35
	*Caltha palustris*	.	.	35
	*Cirsium palustre*	.	.	29
	*Carex hirta*	.	.	29
	*Cirsium oleraceum*	.	.	29
	*Lathyrus pratensis*	.	.	24
	*Lysimachia vulgaris*	.	.	18
	*Lychnis flos-cuculi*	.	.	18
	*Angelica sylvestris*	.	.	18
	**Other species**			
	*Equisetum palustre*	.	50	71
	*Ranunculus repens*	.	50	65
	*Lysimachia nummularia*	66	100	24
	*Veronica beccabunga*	66	.	12
	*Poa palustris*	.	.	24
	*Epilobium parviflorum*	.	.	18
	*Salix purpurea*	.	.	18
	*Galeopsis speciosa*	.	.	18
	*Urtica dioica*	.	.	18
SC	*Carex flava* agg.	.	.	18
	*Juncus inflexus*	.	.	18

**Table 2.** Soil reaction and conductivity in Slovak localities. For location of relevés, see Suppl. material 1

**Relevé number**	4	5	9	10	19
Soil reaction	7.22	7.05	5.26	5.30	7.03
Soil conductivity (μS/cm)	384.9	695.1	867.5	320.0	334.3
