# Supplementary material for: Vegetation affinity of species Typha shuttleworthii in the western part of the Carpathians, with Typhetum shuttleworthii as a new association to Slovakia
Source: Biodivers Data J. 2020 May 4;8:e52151. doi: 10.3897/BDJ.8.e52151 (PMC7217979; doi:10.3897/BDJ.8.e52151)
Supplement: Supplementary material 1 — Shortened full table of phytosociological relevés [file bdj-08-e52151-s001.doc]

Shortened table of phytosociological relevés with the occurrence of *Typha shuttleworthii* in the Western Carpathians and the adjacent part of the Eastern Carpathians (CZ – Czech Republic, PL – Poland, SK – Slovakia, UA – Ukraine); relevés of the association *Typhetum shuttleworthii* are shaded. Only species with occurrence in at least 3 relevés are presented. Unpublished Slovak relevés with all plant taxa are stored in national databases [GIVD](http://www.botanik.uni-greifswald.de/558.html) ID: EU-SK-001 (<http://ibot.sav.sk/cdf/>).

MA – *Molinio-Arrhenatheretea*, PM – *Phragmito-Magnocaricetea*, SC – *Scheuchzerio-Caricetea fuscae*

|  | **Relevé number** |  | **1** | **2** | **3** |  | **4** | **5** |  | **6** | **7** | **8** | **9** | **10** | **11** | **12** | **13** | **14** | **15** | **16** | **17** | **18** | **19** | **20** | **21** | **22** |  |
| --- | --- | --- | --- | --- | --- | --- | --- | --- | --- | --- | --- | --- | --- | --- | --- | --- | --- | --- | --- | --- | --- | --- | --- | --- | --- | --- | --- |
|  | **Country** |  | **CZ** | **PL** | **CZ** |  | **SK** | **SK** |  | **PL** | **PL** | **PL** | **SK** | **SK** | **PL** | **SK** | **UA** | **UA** | **UA** | **UA** | **UA** | **PL** | **SK** | **PL** | **PL** | **PL** |  |
|  | **Number of taxa** |  | **4** | **7** | **5** |  | **12** | **17** |  | **11** | **17** | **8** | **29** | **15** | **37** | **38** | **19** | **19** | **16** | **15** | **18** | **17** | **19** | **24** | **9** | **16** |  |
|  | **Number of hidden taxa** |  | **0** | **1** | **0** |  | **0** | **2** |  | **2** | **3** | **1** | **12** | **4** | **21** | **21** | **3** | **6** | **0** | **2** | **3** | **2** | **9** | **8** | **2** | **3** |  |
|  |  |  |  |  |  |  |  |  |  |  |  |  |  |  |  |  |  |  |  |  |  |  |  |  |  |  |  |
|  | *Typha shuttleworthii* | E_1_ | 1 | 2 | 5 |  | 3 | 4 |  | 3 | 1 | 2 | 3 | 1 | 1 | 2 | 4 | 3 | 4 | 4 | 3 | 4 | 4 | 4 | 3 | 2 |  |
|  | **Diagnostic species of the first cluster** | | | | | | | | | | | | | | | | | | | | | | | | | |  |
|  | *Equisetum arvense* | E_1_ | **2** | **.** | **1** |  | . | . |  | . | . | . | . | . | . | . | . | . | . | . | . | . | . | . | . | . |  |
|  | **Diagnostic species of the second cluster** | | | | | | | | | | | | | | | | | | | | | | | | | |  |
| SC | *Calliergonella cuspidata* | E_0_ | . | . | . |  | **3** | **5** |  | . | . | . | . | . | . | . | . | . | . | . | . | . | . | . | . | . |  |
|  | *Drepanocladus aduncus* | E_0_ | . | . | . |  | **2** | **+** |  | . | . | . | . | . | . | . | . | . | . | . | . | . | . | . | . | . |  |
| SC | *Calliergon giganteum* | E_0_ | . | . | . |  | **+** | **+** |  | . | . | . | . | . | . | . | . | . | . | . | . | . | . | . | . | . |  |
|  | *Cardamine hirsuta* | E_1_ | . | . | . |  | **+** | **1** |  | . | . | . | . | . | . | . | . | . | . | . | . | . | . | . | . | . |  |
| PM | *Sparganium erectum* | E_1_ | . | 1 | . |  | **+** | **+** |  | . | . | . | . | . | . | . | . | + | . | . | . | . | . | . | . | . |  |
|  | *Juncus articulatus* | E_1_ | . | . | . |  | **+** | **1** |  | . | . | . | . | . | 1 | . | . | . | . | . | . | 2 | + | . | . | . |  |
| PM | *Carex rostrata* | E_1_ | . | . | . |  | **5** | **+** |  | . | . | 2 | . | 4 | . | . | 1 | . | . | . | . | . | . | . | . | . |  |
|  | **Diagnostic taxa of the third cluster** | | |  |  |  |  |  |  |  |  |  |  |  |  |  |  |  |  |  |  |  |  |  |  |  |  |
| PM | *Juncus effusus* | E_1_ | . | . | . |  | . | . |  | **1** | **1** | **1** | **3** | **3** | **3** | **.** | **1** | **.** | **+** | **+** | **+** | **.** | **1** | **1** | . | **.** |  |
| PM | *Lycopus europaeus* | E_1_ | . | . | . |  | . | . |  | **.** | **1** | **.** | **+** | **+** | **+** | **1** | **+** | **+** | **+** | **+** | **.** | **+** | **.** | **1** | . | **.** |  |
| MA | *Scirpus sylvaticus* | E_1_ | . | . | . |  | . | . |  | 1 | 5 | 4 | + | . | 2 | 2 | 2 | . | + | + | . | . | . | . | 1 | . |  |
| PM,MA | *Lythrum salicaria* | E_1_ | . | . | . |  | . | . |  | **+** | **1** | **2** | **+** | **2** | **+** | **1** | **.** | **.** | **.** | **+** | **.** | **.** | **1** | **.** | . | **.** |  |
|  | ***Phragmito-Magnocariceta*** | |  |  |  |  |  |  |  |  |  |  |  |  |  |  |  |  |  |  |  |  |  |  |  |  |  |
|  | *Galium palustre* | E_1_ | . | . | . |  | + | 2 |  | . | . | . | . | 1 | . | 1 | 2 | . | 1 | + | + | 1 | . | . | . | . |  |
|  | *Typha latifolia* | E_1_ | 5 | . | . |  | . | + |  | . | . | + | 2 | . | . | . | . | . | . | . | . | 1 | 1 | . | 2 | 1 |  |
|  | *Epilobium hirsutum* | E_1_ | . | . | . |  | . | . |  | **+** | **+** | **.** | **+** | **+** | **.** | **1** | **+** | **+** | **.** | **.** | **+** | **.** | **.** | **.** | . | **.** |  |
|  | *Glyceria notata* | E_1_ | . | 1 | . |  | . | . |  | . | . | . | . | . | 1 | . | . | + | . | . | + | 3 | 1 | . | . | . |  |
|  | *Eleocharis palustris* agg. | E_1_ | . | . | . |  | + | 3 |  | . | . | . | . | . | . | . | + | + | . | + | + | . | . | . | . | . |  |
|  | *Alisma plantago-aquatica* | E_1_ | . | + | . |  | . | . |  | . | . | . | . | . | . | . | + | + | . | . | . | . | . | . | . | . |  |
|  | ***Molinio-Arrhenatheretea*** |  |  |  |  |  |  |  |  |  |  |  |  |  |  |  |  |  |  |  |  |  |  |  |  |  |  |
|  | *Mentha longifolia* | E_1_ | . | + | 2 |  | . | . |  | 2 | 1 | . | . | . | . | 2 | + | + | + | + | + | 1 | . | 1 | 1 | 3 | |
|  | *Myosotis palustris* agg. | E_1_ | . | . | . |  | r | + |  | . | 1 | . | + | . | 2 | 2 | + | . | + | + | . | . | . | 1 | . | 1 | |
|  | *Agrostis stolonifera* agg. | E_1_ | . | . | . |  | . | . |  | . | . | . | . | . | 1 | . | 1 | 1 | 1 | . | . | + | + | + | . | + |  |
|  | *Filipendula ulmaria* | E_1_ | . | . | . |  | . | . |  | + | . | + | . | . | + | 1 | + | . | . | . | + | . | . | . | . | + |  |
|  | *Galium rivale* | E_1_ | . | . | . |  | . | . |  | 1 | 1 | . | . | . | 1 | . | . | . | . | . | . | + | . | 2 | . | 3 |  |
|  | *Caltha palustris* | E_1_ | . | . | . |  | . | . |  | . | . | . | . | . | . | 2 | + | + | + | . | . | 1 | . | 2 | . | . |  |
|  | *Cirsium palustre* | E_1_ | . | . | . |  | . | . |  | . | . | . | 1 | 1 | . | 1 | . | . | . | . | . | . | . | + | 1 | . |  |
|  | *Carex hirta* | E_1_ | . | . | . |  | . | . |  | . | . | . | + | . | . | 1 | . | + | + | . | + | . | . | . | . | . |  |
|  | *Cirsium oleraceum* | E_1_ | . | . | . |  | . | . |  | . | . | . | . | . | . | . | . | + | + | . | + | . | . | 1 | . | 1 |  |
|  | *Lathyrus pratensis* | E_1_ | . | . | . |  | . | . |  | . | . | . | . | . | 2 | 2 | . | . | . | . | . | . | . | + | . | 1 |  |
|  | *Lysimachia vulgaris* | E_1_ | . | . | . |  | . | . |  | . | . | . | + | . | . | 2 | . | . | . | . | . | . | 1 | . | . | . |  |
|  | *Lychnis flos-cuculi* | E_1_ | . | . | . |  | . | . |  | . | . | . | + | . | . | 1 | + | . | . | . | . | . | . | . | . | . |  |
|  | *Angelica sylvestris* | E_1_ | . | . | . |  | . | . |  | . | . | . | r | . | . | . | . | . | . | . | . | . | . | + | . | + |  |
|  | **Other species** |  |  |  |  |  |  |  |  |  |  |  |  |  |  |  |  |  |  |  |  |  |  |  |  |  |  |
|  | *Equisetum palustre* | E_1_ | . | . | . |  | . | 1 |  | + | 1 | . | 1 | 2 | . | 3 | . | . | + | + | + | 2 | . | 1 | 2 | + |  |
|  | *Ranunculus repens* | E_1_ | . | . | . |  | . | + |  | . | + | . | + | . | 1 | . | 1 | . | 1 | 2 | + | 2 | + | 1 | . | + |  |
|  | *Lysimachia nummularia* | E_1_ | 1 | . | + |  | + | + |  | . | . | . | . | . | . | . | . | . | 1 | + | + | 1 | . | . | . | . |  |
|  | *Veronica beccabunga* | E_1_ | . | 1 | 1 |  | . | . |  | . | . | . | . | . | . | . | . | 1 | . | . | . | 1 | . | . | . | . |  |
|  | *Poa palustris* | E_1_ | . | . | . |  | . | . |  | . | + | . | 1 | + | . | . | . | . | 1 | . | . | . | . | . | . | . |  |
|  | *Epilobium parviflorum* | E_1_ | . | . | . |  | . | . |  | . | . | . | . | . | 1 | . | . | . | . | . | . | 1 | . | . | + | . |  |
|  | *Salix purpurea* | E_1_ | . | . | . |  | . | . |  | . | + | . | . | . | . | . | . | . | . | . | 1 | . | + | . | . | . |  |
|  | *Galeopsis speciosa* | E_1_ | . | . | . |  | . | . |  | . | + | . | . | . | . | . | . | . | . | . | . | . | . | + | . | 1 |  |
|  | *Urtica dioica* | E_1_ | . | . | . |  | . | . |  | . | . | . | + | + | + | . | . | . | . | . | . | . | . | . | . | . |  |
| SC | *Carex flava* agg. | E_1_ | . | . | . |  | . | . |  | . | . | . | . | . | + | 2 | . | . | . | + | . | . | . | . | . | . |  |
|  | *Juncus inflexus* | E_1_ | . | . | . |  | . | . |  | . | . | . | . | . | . | . | . | . | + | . | 1 | . | . | 1 | . | . |  |

**Localities of relevés:**

**1** – Bartošová et al. (2008), tab. 2, rel. 19; **2** – Kozłowska et al. (2011), tab. 2, rel. 4; **3** – Bartošová et al. (2008), tab. 2, rel. 29; **4** – Muránska planina Mts, Červená Skala village, Salašná, 48º48ˊ11.1˝N, 20º7ˊ15.6˝E, 825 m a.s.l., mean annual temperature 6.1°C, total annual precipitation 1078 mm, carst pool, area 24 m^2^, cover E_1_ 95%, cover E_0_ 40%, author: D. Blanár, date: 24. 8. 2018; **5** – Muránska planina Mts, Červená Skala village, Salašná, 48º48ˊ11.1˝N, 20º7ˊ15.6˝E, 825 m a.s.l., 6.1°C, 1078 mm, carst pool, area 24 m^2^, cover E_1_ 85%, cover E_0_ 95%, author: D. Blanár, date: 24. 8. 2018; **6** – Kozłowska et al. (2011), tab. 2, rel. 6; **7** – Kozłowska et al. (2011), tab. 2, rel. 9; **8** – Kozłowska et al. (2011), tab. 2, rel. 8; **9** – Oravská kotlina basin, Bobrov village, 1.5 m e. from the village, alluvium of the Poľanovský potok stream, 49º26ˊ34.9˝N, 19º34ˊ20.8˝E, 609 m a.s.l., 6.9 °C, 1123 mm, terrain depression, area 16 m^2^, cover E_1_ 100%, cover E_0_ 20%, author: R. Hrivnák, M. Slezák, date: 2. 8. 2016; **10** – Oravská kotlina basin, Bobrov village, 1.5 m e. from the village, alluvium of the Poľanovský potok stream, 49º26ˊ36.8˝N, 19º34ˊ16.8˝E, 609 m a.s.l., 6.9 °C, 1123 mm, terrain depression, area 16 m^2^, cover E_1_ 100%, cover E_0_ 1%, author: R. Hrivnák, M. Slezák, date: 2. 8. 2016; **11** – Kozłowska et al. (2011), tab. 2, rel. 7; **12** – Uhrin & Bača (2005), page 105; **13** – Borsukevych (2011), table, rel. 2; **14** – Borsukevych (2011), table, rel. 4; **15** – Borsukevych (2011), table, rel. 5; **16** – Borsukevych (2011), table, rel. 3; **17** – Borsukevych (2011), table, rel. 1; **18** – Kozłowska et al. (2011), tab. 2, rel. 1; **19** – Horehronské podolie basin, Rohozná village, 48º47ˊ28.8˝N, 19º43ˊ20.2˝E, 539 m a.s.l., 7.3 °C, 1170 mm, shallow terrain depression, area 9 m^2^, cover E_1_ 70%, cover E_0_ 1%, author: R. Hrivnák, date: 24. 7. 2018; **20** – Kozłowska et al. (2011), tab. 2, rel. 2; **21** – Kozłowska et al. (2011), tab. 2, rel. 3; **22** – Kozłowska et al. (2011), tab. 2, rel. 5.
