# Supplementary material for: Vegetation affinity of species Typha shuttleworthii in the western part of the Carpathians, with Typhetum shuttleworthii as a new association to Slovakia
Source: Biodivers Data J. 2020 May 4;8:e52151. doi: 10.3897/BDJ.8.e52151 (PMC7217979; doi:10.3897/BDJ.8.e52151)
Supplement: Supplementary material 2 — Unpublished phytosociological relevé [file bdj-08-e52151-s002.docx]

Unpublished phytosociological relevé: Muránska planina Mts, Červená Skala village, Salašná, 48º48ˊ11.1˝N, 20º7ˊ15.6˝E, 825 m a.s.l., carst pool, area 20 m^2^, cover E_1_ 85%, cover E_0_ 5%, author: D. Blanár [bryophytes identified by K. Mišíková], date: 24. 6. 2001, plot ID EU-SK-001-787053.

E_1_: *Carex nigra* 4, *Equisetum palustre* 1, *Carex flava* 1, *Cardamine pratensis* agg. +, *Carex panicea* +, *Deschampsia cespitosa* +, *Eleocharis palustris* agg. +, *Juncus articulatus* +, *J. compressus* +, *Lysimachia nummularia* +, *Mentha arvensis* +, *Potentilla anserina* +, *P. reptans* +, *Ranunculus repens* +, *Triglochin palustre* +, *Typha latifolia* +.

E_0_: *Climacium dendroides* 1, *Bryum pseudotriquetrum* +, *Calliergon giganteum* +, *Plagiomnium elatum* +.
